# Clinical impact of interruption in adjuvant Trastuzumab therapy in patients with operable HER-2 positive breast cancer

**DOI:** 10.1186/s40959-020-00081-9

**Published:** 2020-11-05

**Authors:** Sagar Sardesai, Jasmine Sukumar, Mahmoud Kassem, Marilly Palettas, Julie Stephens, Evan Morgan, Daniel Addison, Ragavendra Baliga, Daniel G. Stover, Jeffrey VanDeusen, Nicole Williams, Mathew Cherian, Maryam Lustberg, Robert Wesolowski, Bhuvaneswari Ramaswamy

**Affiliations:** 1 Stefanie Spielman Comprehensive Breast Cancer, The Ohio State University, Columbus, OH 43210 USA; 2 Division of Medical Oncology, Comprehensive Cancer Center, The Ohio State University Wexner Medical Center, 1204A Lincoln Tower, 1800 Cannon Dr, Columbus, OH USA; 3 Center for Biostatistics, Department of Biomedical Informatics, The Ohio State University, Columbus, OH USA; 4 Division of Cardiology, The Ohio State University, Columbus, OH USA

**Keywords:** HER2, Breast cancer, Trastuzumab, Cardio-oncology, Chemotherapy

## Abstract

**Background:**

Trastuzumab-induced cardiotoxicity (TIC) can lead to early discontinuation of adjuvant therapy, however there is limited evidence on long-term survival outcomes in patients with operable human epidermal growth factor receptor 2 (HER2)-positive breast cancer (BC) experiencing treatment interruption or discontinuation.

**Methods:**

The primary objective of the study was to evaluate disease-free survival (DFS) in non-metastatic, HER2-positive, female BC patients who experienced treatment interruption or early discontinuation of trastuzumab therapy. Clinical and histopathological data were collected on 400 patients at The Ohio State University, an NCI-designated comprehensive cancer center between January 2005 and December 2015. Treatment interruption was defined as any delay of ≥2 weeks during trastuzumab therapy, including permanent cessation prior to completing planned therapy. TIC was defined as LVEF < 50% or > 15 points decline from baseline as evaluated by 2D echocardiogram after initiation of (neo) adjuvant therapy. DFS was defined as the time from diagnosis to first recurrence (loco-regional or distant recurrence) including second primary BC or death. Overall survival (OS) was defined as the time from diagnosis to death or last known follow up. OS/DFS estimates were generated using Kaplan-Meier methods and compared using Log-rank tests. Cox proportional hazard models were used to calculate adjusted hazard ratios (aHR) for OS/DFS.

**Results:**

A total of 369 patients received trastuzumab therapy; 106 (29%) patients experienced treatment interruption at least once and 42 (11%) permanently discontinued trastuzumab prior to completing planned therapy. TIC was the most common reason for interruption (66 patients, 62%). The median duration of trastuzumab in patients with treatment interruption was 11.3 months (range: 0.5–16.9) with 24 (23%) patients receiving ≤6 months of therapy. This duration includes the time delay related to treatment interruption. Patients with any treatment interruption had worse DFS (aHR: 4.4, *p* = 0.001) and OS (aHR: 4.8, *p* < 0.001) after adjusting for age, stage, grade, ER, node status and TIC.

**Conclusions:**

Treatment interruption or early discontinuation of trastuzumab therapy in early HER2-positive BC, most often from TIC, is an independent prognostic marker for worse DFS and OS in operable HER2-positive BC. Future prospective studies should consider targeting at-risk populations and optimizing cardiac function to avoid interruption in trastuzumab therapy.

## Introduction

Breast cancer (BC) is a heterogeneous disease broadly categorized into three distinct phenotypes based on hormone receptor (HR) and human epidermal growth factor receptor 2 (HER2) overexpression [1]. HER2-Neu gene amplification and/or overexpression accounts for 15–20% of all newly diagnosed cases in the United States, which results in an aggressive biology associated with a higher risk of recurrence compared to HR-positive disease [2]. Trastuzumab, a humanized, monoclonal antibody that targets the extracellular domain IV of HER2 receptor, has dramatically changed the prognosis of patients with early invasive HER2-positive disease. Several trials have consistently shown a decrease in risk of BC recurrence and BC-specific mortality with the addition of 1 year of trastuzumab to standard adjuvant chemotherapy [3–6]. Although trastuzumab is well tolerated, it is associated with cardiotoxicity in some patients, which can result in early discontinuation of adjuvant therapy [7]. Trastuzumab-induced cardiotoxicity (TIC) can present clinically with asymptomatic decline in left ventricular ejection fraction (LVEF) (3.2–19%) or symptomatic heart failure (0.5–5.1%) [8–11]. HER2 is expressed in cardiac myocytes and is essential for the preservation of cardiac function and structure [12]. Although this could explain a potential link, the true mechanism of TIC remains unclear [13]. Patients receiving adjuvant trastuzumab undergo periodic monitoring of LVEF every 3 months while on therapy [14]; those experiencing decline in LVEF ≥16% from baseline or LVEF below normal limits and ≥ 10% decline are asked to withhold treatment for at least 4 weeks and continue LVEF monitoring every 4 weeks until resolution. Older age (> 50 years) and pre-existing cardiovascular (CV) diseases, such as uncontrolled hypertension and congestive heart failure, have been implicated in increasing the risk of TIC [10, 15–19]. The incidence of TIC is also higher in patients receiving concomitant or sequential anthracycline (up to 27%) compared to non-anthracycline regimens [4, 20–22]. Several randomized trials have evaluated shorter duration of adjuvant trastuzumab with a goal of minimizing cardiotoxicity while maintaining efficacy of systemic therapy [23–26]. A recent open-label, phase III randomized trial compared 6 months vs. 12 months of adjuvant trastuzumab therapy and showed that shorter duration was non-inferior to standard 1 year of therapy and resulted in less cardiac toxicity. They demonstrated that a four-year disease-free survival (DFS) was 89% (95% CI 88–91) in both arms. The estimated hazard ratio was 1.05 (95% CI 0.88–1.25) indicating non-inferiority (hazard ratio < 1.29) of 6-months of trastuzumab (*p* = 0.01) [23]. On the other hand, several other studies, including a meta-analysis of four trials evaluating shorter duration of adjuvant trastuzumab, failed to report non-inferiority compared to a standard 1 year regimen, cautioning against the adoption of this approach in routine clinical practice [24–26].

While shorter duration of trastuzumab therapy is associated with lower incidence of TIC [23], there is limited evidence to describe disease outcomes in patients with interruption and/or early discontinuation of adjuvant trastuzumab secondary to cardiac toxicity. Interruption or early discontinuation may adversely affect disease outcomes in HER2-positive early BC patients independent of planned duration of adjuvant HER2-directed therapy. Herein, we report the results of a single-institution study assessing survival outcomes in patients who experienced treatment interruption or early discontinuation of adjuvant trastuzumab at The Ohio State University Comprehensive Cancer Center (OSUCCC-James).

## Materials and methods

### Study design

This study was an IRB-approved (OSU 2017C0080) retrospective chart review of clinical and histopathologic data from female patients ≥18 years of age, with HER2-positive, stage I-III BC seen at OSUCCC-James between January 2005 and December 2015. HER2 expression was confirmed per ASCO-CAP guidelines at the time of diagnosis defined by immunohistochemistry of 3+ (uniform, intense membrane staining of > 30% of invasive tumor cells), a fluorescent in situ hybridization (FISH) result of ≥6 HER2 gene copies per nucleus, or a FISH ratio (HER2 gene signals to chromosome 17 centromeric signals) of ≥2.2. Patients with incomplete clinical data, those who had stage IV disease, pathologically in-situ or microinvasive disease at diagnosis (≤1 mm invasive disease in the greatest dimension), and those treated at other institutions were excluded. Treatment interruption was defined as any treatment delay of ≥2 weeks in receiving trastuzumab, including permanent discontinuation prior to completing planned duration of therapy. TIC was defined as LVEF < 50% or > 15 points decline from baseline as evaluated by 2D echocardiogram after initiation of (neo) adjuvant therapy. In concordance with FDA package insert criteria and our institutional guidelines for trastuzumab, patients who experienced a decline in LVEF ≥16% from baseline or LVEF below normal limits and ≥ 10% decline were asked to withhold treatment for at least 4 weeks and referred to a cardiologist to initiate cardioprotective therapy. LVEF monitoring was repeated every 4 weeks until resolution. Trastuzumab re-challenge was only provided to those with a recovery in LVEF to normal.

### Data collection

Data was obtained from The Ohio State University Information Warehouse and uploaded into REDCap database [27]. Missing data were populated using manual review of each patient’s electronic medical record. Data were collected on demographic characteristics, biomarker profiles (ER, PR, and HER2) of the tumor, therapy modalities (surgery, chemotherapy type and regimen, and radiotherapy) and duration of trastuzumab therapy, disease recurrence, and survival outcomes.

### Statistical methods

Demographic and clinical characteristics as well as treatment modalities were summarized using descriptive statistics. DFS was defined as the time from diagnosis to first recurrence (loco-regional or distant recurrence), including second primary BC or death. OS was defined as the time from diagnosis to death or last known follow up. OS/DFS estimates were generated using Kaplan-Meier methods and compared using Log-rank tests. Cox proportional hazard models were used to calculate adjusted hazard ratios (aHR) for OS/DFS. All data analyses were performed using SAS 9.4 (SAS Institute Inc., Cary, NC) or Stata 14 (StataCorp LLC, College Station, TX).

## Results

A total of 400 patients with HER2-positive stage I-III BC were included in this study; 369 (92%) received (neo) adjuvant trastuzumab and were included in survival analyses. Patient demographics, clinical and treatment characteristics of the 400 patients are summarized in Table 1. Patients were predominantly white (83%) and post-menopausal (64%) with a median age of 55 years (range: 26–95) at diagnosis. The majority of patients presented with invasive ductal carcinoma (93%) and grade 3 (61%) disease. Only ten (3%) patients were diagnosed with a lobular histology. One hundred and eighty-six (47%) were diagnosed with node-positive disease and over half received (neo) adjuvant anthracycline (53%). Most common preexisting cardiac risk factors reported in the study were a previous history of smoking (31%), dyslipidemia (33%), or any preexisting cardiac disorders (35%) (Table 1).

**Table 1:** Baseline characteristics of patients

Characteristics	Total (***n*** = 400)
	n (%)
**Age**	
> 50 ys	265 (66%)
< 50 ys	135 (34%)
**BMI**	
> 30	144 (36%)
< 30	256 (64%)
**Race**	
White	331 (83%)
African American	48 (12%)
Other	21 (5%)
**Tumor size**	
< 2 cm	175 (44%)
> 2 cm	225 (56%)
**Nodal Status**	
N0	214 (54%)
N+	186 (47%)
**ER Positive**	
Yes	255 (64%)
No	145 (36%)
**Grade**	
I	15 (4%)
II	137 (34%)
III	245 (61%)
Missing	3 (1%)
**Pre-existing Cardiovascular risk factors**	
Smoking	124 (31%)
Hyperlipidemia	133 (33%)
Hypertension	71 (18%)
Diabetes Mellitus	46 (12%)
Heart Failure	8 (2%)
Ischemic Heart Disease	20 (5%)
Arrhythmias	21 (5%)
Valvular diseases	20 (5%)
**Adjuvant Systemic Therapy**	
**Trastuzumab**	
Yes	369 (92%)
No	31 (8%)
**Chemotherapy**	
Anthracyclines	212 (53%)
Non-Anthracyclines	153 (38%)
No chemotherapy	35 (9%)
**Hormonal Therapy**	
Yes	250 (63%)
No	150 (37%)

A total of 106 (29%) patients experienced trastuzumab interruption at least once for any cause. The baseline demographic and tumor characteristics of the 369 patients who received (neo) adjuvant trastuzumab, categorized by completion or interruption of therapy, is provided in the supplementary table. Table 2 summarizes treatment interruption and subsequent rechallenges of trastuzumab during 1 year of planned adjuvant therapy. A total of 42 (11%) patients permanently stopped trastuzumab before completing planned therapy. Median duration of therapy in patients with any treatment interruption was 11.3 months, and 24 patients (23%) received less than 6 months of trastuzumab therapy. The most common reason for treatment interruption of trastuzumab was cardiotoxicity (*n* = 66, 62%). Treatment interruption resulting from neutropenia (*n* = 16, 15%) during (neo) adjuvant chemotherapy accounts for the majority of non-cardiac events in the study (Table 3). Over half the patients (55%) experiencing any treatment interruption were over 65 years of age. While a higher proportion of patients with treatment interruption reported any anthracycline use, this difference did not reach statistical significance (64% vs. 56%, *p* = 0.150). Seventy-seven (73%) patients experiencing treatment interruption were referred to a cardiologist with a median time from referral to appointment of nine (range: 0–72) days.

**Table 2:** Discontinuation and Re-challenges of Adjuvant Trastuzumab Therapy

Treatment	Number of Patients (%)
**Initial Treatment**	369
Completed planned therapy	263 (71%)
First Discontinuation	106 (29%)
Permanently Discontinued	29 (27%)
**First Re-challenge**	77 (73%)
Completed therapy	55 (71%)
Second Discontinuation	22 (29%)
Permanently Discontinued	12 (16%)
**Second Re-challenge**	10 (45.5%)
Completed therapy	9 (90%)
Third Discontinuation	1 (10%)
Permanently Discontinued	1 (10%)
**Total Permanently Discontinued**	42 (11%)

**Table 3:** Causes of Trastuzumab Discontinuation

Adverse events	Total (***n*** = 106)
	n (%)
Cardiomyopahy	66 (62%)
Neutropenia	16 (15%)
Disease Progression	5 (5%)
Patient Preference	4 (4%)
Thrombocytopenia	3 (3%)
Shortness of Breath	2 (2%)
Others	10 (9%)

**Fig. 1:**
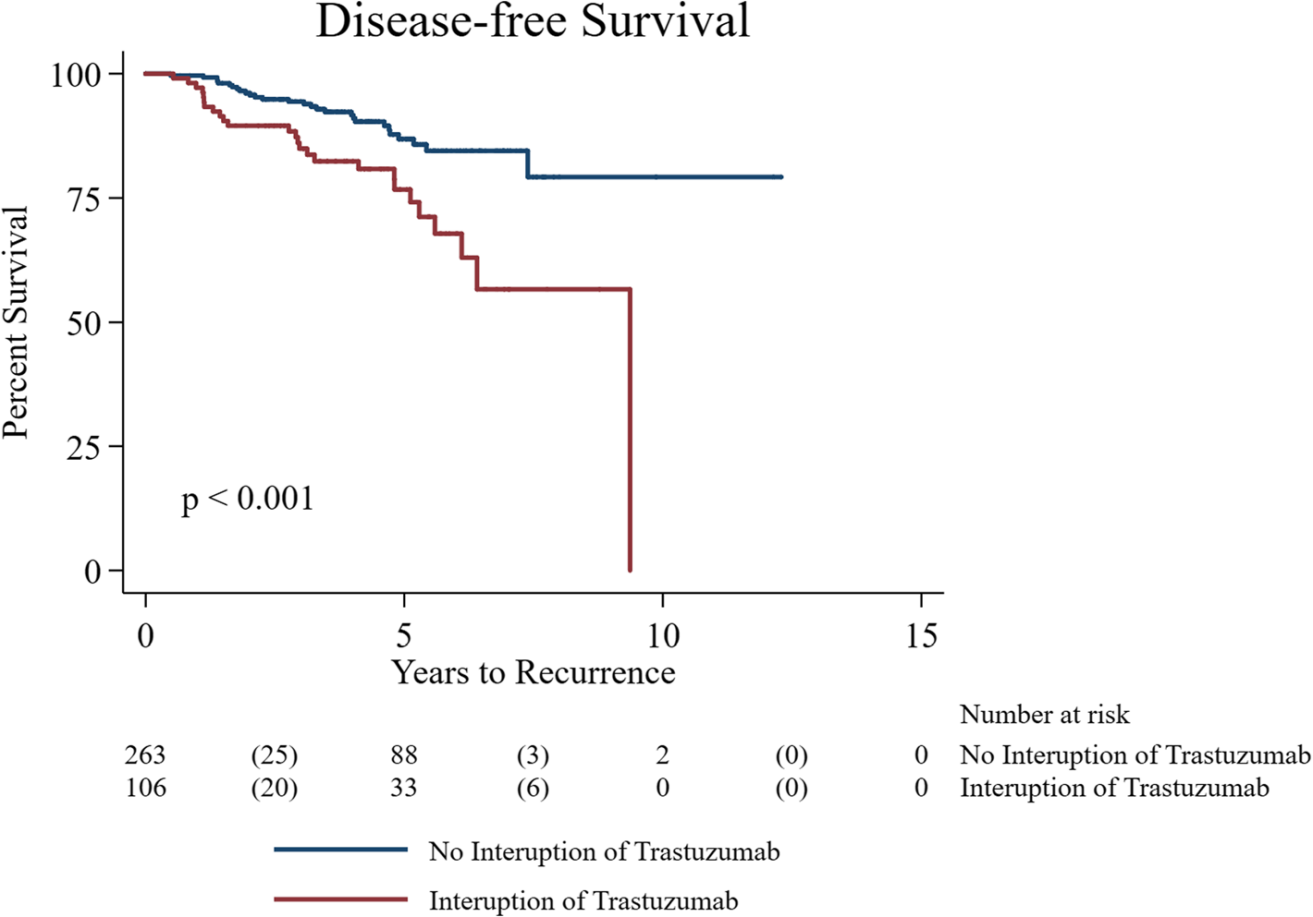
Kaplan Meier curve showing the disease free survival between patients on trastuzumab and patients who had interruption (unadjusted log-rank results)

**Fig. 2:**
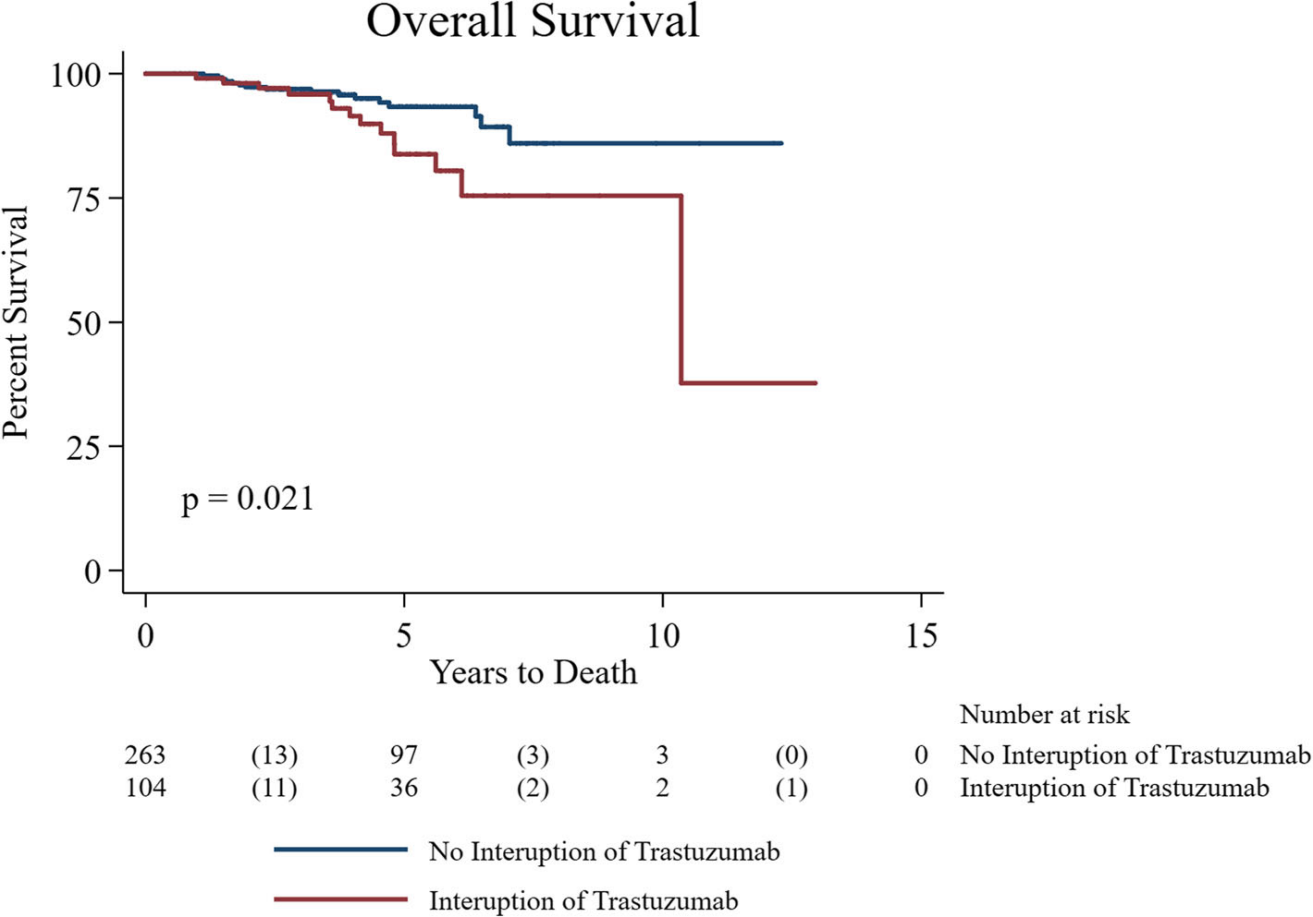
Kaplan Meier curve showing the overall survival between patients on trastuzumab and patients who had interruption (unadjusted log-rank results)

Unadjusted Log-rank tests revealed patients who experienced any interruption in trastuzumab therapy compared to those who had no interruption had worse DFS and OS (*p* = 0.001 and *p* = 0.021, respectively) (Figs. 1 and 2). Multivariable analyses confirmed significant worse DFS (aHR: 4.4, 95% CI: 1.8–10.5, *p* = 0.001) and OS (aHR: 4.8, 95% CI: 2.5–9.2, *p* < 0.001) after adjusting for age, stage, grade, ER, node status and TIC (Tables 4 and 5). A total of 208 (56%) patients received beta blockers (BB) and/or angiotensin converting enzyme inhibitor (ACEi) therapy prior to or after BC diagnosis. The use of cardioprotective therapy (BB and/or ACEi) was significantly higher in patients with known TIC (89% vs. 11%; *p* < 0.001). Patients receiving cardioprotective therapy showed worse OS compared to those not on cardioprotective therapy (HR: 2.7, 95% CI: 1.3–5.8, *p* = 0.010).

**Table 4:** Multivariate analysis of hazard ratio of DFS with trastuzumab discontinuation

Variable	Adjusted Hazard Ratio	Std. Err.	95% CI	***P***-value
**Discontinuation Trastuzumab**	4.38	1.95	1.83–10.46	0.001
**Age**	0.99	0.02	0.97–1.03	0.750
**Stage, 1 or 2 vs Stage 3**	3.62	1.59	1.53–8.59	0.003
**Grade, 1 or 2 vs Grade 3**	1.39	0.56	0.63–3.06	0.416
**ER status, Negative vs Positive**	0.60	0.26	0.25–1.42	0.242
**Cardiomyopathy, Yes vs No**	0.25	0.14	0.08–0.75	0.014

**Table 5:** Multivariate analysis of hazard ratio of OS with trastuzumab discontinuation

Variable	Adjusted Hazard Ratio	Std. Err.	95% CI	***P***-value
**Discontinuation Trastuzumab**	4.76	1.59	2.47–9.16	< 0.001
**Age**	0.99	0.01	0.97–1.01	0.302
**Stage, 1 or 2 vs Stage 3**	2.45	0.74	1.36–4.41	0.003
**Grade, 1 or 2 vs Grade 3**	1.69	0.53	0.91–3.14	0.094
**ER status, Negative vs Positive**	0.64	0.20	0.34–1.20	0.163
**Cardiomyopathy Yes vs No**	0.31	0.13	0.14–0.70	0.004

## Discussion

This contemporary population-based study in 369 consecutive female patients receiving trastuzumab for early stage HER-2 positive BC between January 2005 and December 2015 showed that 106 (29%) patients experienced treatment interruption of ≥2 weeks, among whom 42 (11%) experienced permanent discontinuation of trastuzumab therapy, largely due to cardiac toxicity.

Evidence from randomized trials with adjuvant trastuzumab in combination with an anthracycline –taxane-based regimen have shown a 12–15% asymptomatic decline in LVEF with very few patients reporting permanent discontinuation [10, 11, 28–30]. For instance, secondary analyses of cardiac events in NSABP B31 showed a meager 4% discontinuation of adjuvant trastuzumab [11]. Careful selection of patients with limited to no medical comorbidities in clinical trials is imperative given the concern for long-term toxicity in a curative intent patient population. However, clinicians routinely have to make complex medical decisions weighing the potential benefit of HER2-directed therapy against risks of pre-existing comorbidities in everyday practice. In addition, minority and elderly patents are often underrepresented in therapeutic studies. Our study highlights this ‘real world’ disparity with a significantly higher proportion of patients with treatment interruption or permanent cessation of trastuzumab therapy for early HER2-positive BC, when only half received an anthracycline-based regimen. These results are consistent with other large retrospective series showing higher overall discontinuation of adjuvant trastuzumab in clinical practice, with Wang et al. reporting up to 41% of patients discontinuing planned therapy, most events as a result of TIC [31, 32]. However, a majority of patients reported by Wang et al. were elderly (mean age 71.6 years), emphasizing the need for further investigation of risk factors and risk mitigation strategies in this patient population [33].

Approximately 18% of all patients in the study experienced TIC leading to treatment delays. Consistent with previous studies, TIC was responsible for the majority of treatment interruption (62%) with adjuvant trastuzumab [31, 34, 35]. Shorter (< 6 months) planned duration of adjuvant trastuzumab has been studied in an attempt to reduce risk of CV events while maintaining efficacy of targeted therapy [23, 24, 36]. While 6 months of adjuvant targeted therapy is associated with favorable CV risk, two major phase III randomized adjuvant trials reported conflicting results on non-inferiority in invasive DFS with shorter duration to standard 1 year of therapy [23, 24]. Secondary analyses of US PHARE study revealed that metastases-free survival, a key endpoint, was significantly worse with 6 months vs. 1 year of therapy. However, long term outcomes, especially in patients experiencing treatment interruptions or discontinuation, are lacking from large prospective studies given the small number of events. Our analyses suggest any interruption in trastuzumab treatment as a prognostic factor with up to three times worse DFS and OS compared to patients who finished planned 1 year of therapy or had < 14 days of interruption in treatment. Multivariable analyses confirmed this effect after adjusting for major confounders, including drug-induced cardiotoxicity, indicating that worse outcomes are likely related to cancer recurrence in these patients.

Similar concerns have been reported with early trastuzumab interruption in other studies as well [31–33, 37]. Gong et al. reported higher risk of disease recurrence in patients stopping adjuvant trastuzumab and emphasized that early discontinuation is a stronger prognostic factor for worse survival than cardiotoxicity in this patient population. As in our study, they also reported a high proportion of patients completing therapy with over 85% patients receiving ≥16 doses [32]. Additionally, a retrospective analysis of 5547 patients who received adjuvant trastuzumab demonstrated a significantly higher risk of BC relapse and death in patients who did not complete adjuvant trastuzumab. The 5 year DFS and OS was 94 and 95%, respectively, in those who completed full treatment compared with 80% (HR 3.15; *p* < 0.001) and 84% (HR 2.12; *p* = 0.005), respectively, in patients who received treatment interruption in relation to a cardiac event within the treatment period [37, 38]. These results taken together suggest a strong need for early assessment and optimization of CV status of at-risk patients to minimize any interruption in targeted therapy.

Recent studies demonstrate the safety of continuing trastuzumab with mild cardiac dysfunction in the setting of collaborative management with cardiology. In the present study, TIC was defined based on the FDA prescription instructions [14], which recommend to hold therapy for at least 4 weeks in those experiencing a decline in LVEF ≥16% from baseline or if LVEF falls ≥10% below baseline and below the lower limit of normal. Trastuzumab can subsequently be restarted with resolution of cardiac dysfunction. However, since completion of this study, two = single arm prospective trials, SCHOLAR [39] and SAFE-HEaRt [22], have investigated the continuation HER2-targeted therapy while concomitantly starting cardioprotective therapy in patients who develop mildly reduced LVEF. Although larger and randomized trials are needed, both of these studies demonstrate safety data with lack of further deterioration in cardiac function in approximately 90% of cases. In line with this, ESMO has released new guidelines in 2020 [40] which recommend to consider continuation of trastuzumab with initiation of cardioprotective therapy as an option in patients with mild asymptomatic decline in LVEF (level of evidence III, grade of recommendation A). This is defined as an LVEF decrease ≥10% from baseline or a drop in LVEF to ≥40% but < 50%. Given the considerable negative impact of treatment interruption on breast cancer recurrence and survival as demonstrated in our study and others [31–33, 37], we recommend reconsideration of trastuzumab package insert criteria for drug interruption based on mild asymptomatic heart failure. Application of this clinical practice may be offered as an option in patients based on shared decision making between oncologist, cardiologist and patient of risks vs benefits in those who can receive close monitoring.

Cardioprotective therapy, including BB and ACEi, have a well-documented safety profile and are commonly used in practice to improve cardiac outcomes in patients with preexisting heart disease. Current evidence suggests that they may be crucial in preventing treatment interruption of adjuvant trastuzumab in HER2-positive BC patients [41, 42]. In a randomized, double-blinded placebo-controlled trial, Guglin et al. showed that cardiotoxicity-free survival was higher for both lisinopril (HR: 0.53;*p* = 0.015), and carvedilol (HR: 0.49; *p* = 0.009) compared to placebo with fewer treatment interruptions in the intervention group [41]. While we report worse OS in patients on cardioprotective therapy, this likely results from a higher proportion of patients receiving BB and/or ACEi therapy for other concurrent illnesses (72%) that may adversely impact survival.

The use of BB and ACEi prior to initiating anti-HER2 therapy needs to be carefully weighed against unintended consequences, such as rising health care costs, over-diagnosis or over-treatment associated with the increased use of cardiac surveillance and prophylactic therapy [43]. Close collaboration between oncologists and cardiologists is needed to guide personalized therapy for vulnerable patients [39, 44–46]. Our institutional guidelines encourage referral to cardio-oncology for at–risk patients prior to initiating trastuzumab therapy and early intervention in patients who experience LVEF decline while on targeted therapy. A majority of patients who experienced treatment interruption (73%) in this study were referred and received timely evaluation by cardio-oncology.

Another strategy to reduce cardiac risk has been to employ non-anthracycline regimens in (neo) adjuvant therapy [47, 48]. Our analyses did not show a significant difference in the use of anthracycline in patients who experienced any treatment interruption compared to controls. This is likely due to careful selection of patients deemed fit to receive adjuvant anthracycline. The advent of adjuvant TDM1 for patients with high risk residual disease and dual anti-HER2 therapies, such as combination with pertuzumab, will further impact the use of adjuvant anthracycline in these patients [49, 50].

Our study is not without limitations. While we have a detailed account of treatment data, reason for trastuzumab interruption/discontinuation, known pre-existing cardiac comorbidities, rechallenge rates and use of cardioprotective therapy, the study is limited by its retrospective nature, including possible selection bias that may impact outcome assessment. Dose titration was at the discretion of the treating clinicians. Additionally, it is a single-institution study with a limited number of events. Given the retrospective nature of chart review, it was challenging to identify time of initiation (before or after onset of TIC) and treatment indication (TIC vs. concurrent cardiac illness) for the use of BB and/or ACEi therapy in patients. This made it difficult to interpret the association of the use of cardioprotective therapy on the incidence of TIC and treatment interruption of trastuzumab. Lastly, our observational study sought to evaluate the association of treatment interruption in adjuvant trastuzumab with long-term cancer outcomes so as to augment patient care and survivorship, but cannot confirm causality.

## Conclusion

In summary, treatment interruption or early discontinuation of trastuzumab therapy in early HER2-positive BC is associated with worse DFS and OS irrespective of age, receptor status, stage of disease and treatment-induced cardiotoxicity. The mechanisms and optimal timing of cardioprotective medications as well as best practices in treatment interruption in patients receiving adjuvant trastuzumab therapy requires further investigation in the modern era of cardio-oncology. Growing cooperation between oncologists and cardiologists can foster development of consensus-based guidelines for surveillance, prevention, and care of individuals initiating HER2-directed therapy in BC.

## Supplementary Information

**Additional file 1: Supplementary Table.** Baseline characteristics of patients who received trastuzumab, categorized by completion vs interruption of therapy.

## Data Availability

The data that support the findings of this study are available from The Ohio State University Information Warehouse but restrictions apply to the availability of these data, which were used under license for the current study, and so are not publicly available. However, data are available from the authors upon reasonable request and with permission of The Ohio State University IRB.

## Abbreviations

ACEi: Angiotensin converting enzyme inhibitor
aHR: Adjusted Hazards Ratio
BB: Beta blocker
BC: Breast cancer
DFS: Disease-free survival
FISH: Fluorescence in situ hybridization
HER2: Human epidermal growth factor receptor 2
HR: Hormone receptor
LVEF: Left ventricular ejection fraction
OS: Overall survival
TIC: Trastuzumab-induced cardiomyopathy
