# Supplementary material for: Clinical impact of interruption in adjuvant Trastuzumab therapy in patients with operable HER-2 positive breast cancer
Source: Cardiooncology. 2020 Nov 5;6:26. doi: 10.1186/s40959-020-00081-9 (PMC7643282; doi:10.1186/s40959-020-00081-9)
Supplement: Supplementary file 1 — Additional file 1: Supplementary Table. Baseline characteristics of patients who received trastuzumab, categorized by completion vs interruption of therapy. [file 40959_2020_81_MOESM1_ESM.docx]

**Supplementary Table:**

**Baseline characteristics of patients who received trastuzumab, categorized by completion vs interruption of therapy**

| **Characteristics** | **Completion of Trastuzumab**  **n= 263** | **Interruption of Trastuzumab**  **n= 106** | **P-value** |
| --- | --- | --- | --- |
|  | **n (%)** | **n (%)** |  |
| **Age** |  |  | 0.984 |
| > 50 ys | 169 (64%) | 68 (64%) |  |
| < 50 ys | 94 (36%) | 38 (36%) |  |
| **BMI** |  |  | 0.639 |
| >30 | 90 (34%) | 39 (37%) |  |
| < 30 | 173 (66%) | 67 (63%) |  |
| **Race** |  |  | 0.159 |
| White | 224 (85%) | 83 (78%) |  |
| African American | 24 (9%) | 17 (16%) |  |
| Other | 15 (6%) | 6 (6%) |  |
| **Tumor size** |  |  | 0.001 |
| <2 cm | 122 (46%) | 29 (27%) |  |
| >2 cm | 141 (54%) | 77(73%) |  |
| **Nodal Status** |  |  | 0.278 |
| N0 | 138 (52%) | 49 (46%) |  |
| N+ | 125 (48%) | 57 (54%) |  |
| **ER Positive** |  |  | 0.189 |
| Yes | 162 (62%) | 73 (69%) |  |
| No | 101 (38%) | 33 (31%) |  |
| **Grade** |  |  | 0.292 |
| I | 10 (4%) | 4 (4%) |  |
| II | 93 (35%) | 29 (27%) |  |
| III | 157 (60%) | 73 (69%) |  |
| Missing | 3 (1%) | 0 (0%) |  |
